# 纯/半实性GGN的诊断并非易事

**DOI:** 10.3779/j.issn.1009-3419.2018.03.14

**Published:** 2018-03-20

**Authors:** 晓菁 赵

**Affiliations:** 上海交通大学附属仁济医院胸外科 Department of Toracic Surgery, Renji Hospital Afliated to Shanghai Jiao Tong University

近年来随着影像学技术的发展和低剂量螺旋计算机断层扫描(computed tomography, CT)体检的普及，肺磨玻璃样结节(ground-glass nodule, GGN)的检出率逐年升高。临床上持续存在的肺GGN为早期肺腺癌或癌前病变的可能性较大，包括：非典型腺瘤样增生(atypical adenomatous hy perplasia, AAH)、原位腺癌(adenocarcinoma *in situ*, AIS)、微浸润腺癌(minimally invasive carcinoma, MIA)及浸润性腺癌(invasive carcinoma, IA)^[1]^。

根据新分类，肺腺癌各亚型侵袭性逐渐增强，预后差异性显著，而临床上对于表现为GGN的良性病变和早期肺腺癌各亚型的处理方法有着较大差异^[2]^。良性GGN仅需密切观察，而早期肺腺癌的浸润前病变(AAH和AIS)不会出现淋巴结及血行转移，此类病变可以密切随访或仅做计划性局部切除，淋巴结仅需要采样，而不需要进行系统性清扫，5年生存率可以达到100%。MIA是介于PL和IA之间的病变，本身无局部淋巴结转移及远处血行转移，但较易进展为IA而导致转移的发生，因此，对MIA需要及时手术，可以根据肿瘤大小、磨玻璃成分等进行亚肺叶切除或肺叶切除，并进行系统性淋巴结清扫或采样，及时手术切除的MIA 5年生存率接近100%。而表现为GGN的IA患者，易发生淋巴结转移及血行远处转移，因此，除部分≤2 cm且以磨玻璃成分为主(≥75%)病灶可以进行亚肺叶切除外，手术不但需要进行肺叶切除还要进行系统性淋巴结清扫，5年生存率最高仅能达到60%-80%。另外，对表现为GGN的浸润前病变及部分较小的MIA进行局部切除或解剖性肺段切除，可以在保证肿瘤切除原则的基础上最大限度的保留肺功能，这对提高患者，特别是老年患者术后生活质量至关重要。

因此，术前区分GGN的良恶性及恶性侵袭性程度，并由此鉴别出表现为GGN的早期肺腺癌各亚型具有重要意义，既可以避免过度医疗，防止不必要的手术，也是制定个体化治疗方案的重要依据。

目前的临床研究主要集中于在薄层CT成像中测量GGN的大小^[3]^、CT值^[4, 5]^、实性成分大小及其在GGN中所占比例(C/T比值)^[6]^等来区分GGN的各亚型及其侵袭性，希望在影像学发现和组织病理学之间建立联系，从而实现无创、定量、客观的评估，也已经取得一定的成绩。然而，目前仍有很多问题困扰着研究的进展。

影像学方面：(1)GGN各亚型之间的肿瘤大小、CT值范围重叠现象严重，虽然它们的平均值可以有统计学显著性差异，但实际上各亚型之间区分度是不够的；(2) mGGN中对实性成分的定义缺乏量化标准，仅凭各自的主观判断，影响其精确度；(3)尚无好的工具及方法来精确测量mGGN中的实性成分，虽然UICC第8版肺癌分期把实性成分的大小用于临床T分期^[7]^，但临床实际应用受到限制；(4)mGGN中的实性成分除肿瘤细胞团外，还包括疤痕、肺泡塌陷、巨噬细胞在肺泡中聚集、肺泡内出血等，还无法通过影像学进行精确区分；(5)有序的肺泡样或乳头样为主的IA也常表现为无显著实密成分的pGGN；(6)各研究中心得出的肺GGN各亚型的阈值并不一致，甚至相差较大，无法形成标准用于临床。因此，我们尚无法通过目前的研究方法对GGN各亚型进行精确区分。

病理学方面：正确的病理学诊断是进行GGN影像学研究的基础，然而，然而目前对GGN的诊断存在困扰。(1)取材：目前常规病理取材只能取GGN的一部分，而由于肿瘤异质性的存在，不能反映肿瘤的全貌；术后石蜡病理标本因术中冰冻病理切片的取材而部分缺失，尤其对于实性成分较小的GGN，可能由于术中冰冻切片而出现术后石蜡标本切片时已无实性成分的可能，导致诊断误差。(2)读片：虽然世界卫生组织(World Health Organization, WHO)对诊断AAH、AIS、MIA、IA有典型的图谱标准，然而临床实际应用中仅有20%的病理图片是典型的，近80%的是不典型的，要靠病理科主观判断来进行诊断，从而导致诊断误差。正常情况下，临床病理结果是指导临床医生进行外科手术的依据。与实性肿块不一样，肿瘤的异质性在mGGN的诊断中尤为突出。

由于目前肺GGN的病理诊断精度不够及相关临床研究中诸多问题一直困扰着我们，而这些现象的发生主要与肿瘤的异质性相关，肿瘤异质性是与恶性肿瘤侵袭性及反映疗效等相关的重要生物学特征，它包含了肿瘤内部的代谢活性、细胞密度、脉管分布、组织坏死等组织特点，在CT影像中体现为结节内二维纹理的变化；因为肺结节是三维的，二维纹理分析必然丢失大量信息。日本JCOG0804研究结果表明，对直径≤2 cm的肺GGN，无论是AIS、MIA，还是IA，在GGN中磨玻璃成分≥75%(C/T值≤25%)的情况下，亚肺叶切除(楔形切除或解剖性肺段切除)与肺叶切除的远期疗效相似。

这些都提示我们是否需要换一种思路去研究、解决问题？如在病理诊断方面是否可以通过根据CT片上GGN特征进行精确病理取材切片来达到精确病理诊断的目的？而在临床肺GGN影像学研究方面，国内上海市胸科医院胸外科方文涛教授研究小组取得了一定的进展^[8]^，他们研究发现，使用随机森林算法预测肺GGN良恶性的诊断准确率达95.1%，而预测肺GGN侵袭性的诊断准确率达到83%。然而该研究的本质是用病历和CT图像中提取的结构化特征做模型，优点是结合了病历数据和影像特征，从多模态角度进行预测，比单一看影像要准确；缺点是增加了大量的工作量，也无法避免因主观判断而产生的偏差，而且这种方法并不能直接进行自动图像识别，仍然依赖于医生读片并描述特征。得益于方教授的思路，我们是否可以采用更智能化的、能够自动学习、自动获取多维图像特征进行图像识别的人工智能技术，或者更深入的对能够更好地反映肿瘤异质性的三维肺图像纹理分析技术进行研究^[9]^，来达到我们应用影像学方法精确预测肺GGN良恶性及侵袭性并指导临床工作的目的？这都是值得我们胸外科医生以及与本科相关的病理科、影像科医生思考的问题，值得我们去探索。

## References

[1] Travis WD, Brambilla E, Noguchi M (2011). International Association for the Study of Lung Cancer/American Thoracic Society/European Respiratory Society: international multidisciplinary classification of lung adenocarcinoma: executive summary. Proc Am Thorac Soc.

[2] Lee HJ, Kim YT, Kang CH (2013). Epidermal growth factor receptor mutation in lung adenocarcinomas: relationship with CT characteristics and histologic subtypes. Radiology.

[3] Lee SM, Park CM, Goo JM (2013). Invasive pulmonary adenocarcinomas versus preinvasive lesions appearing as ground-glass nodules: differentiation by using CT features. Radiology.

[4] Lim HJ, Ahn S, Lee KS (2013). Persistent pure ground glass opacity lung nodules >/= 10 mm in diameter at CT scan: histopathologic comparisons and prognostic implications. Chest.

[5] Tamura M, Matsumoto I, Saito D (2017). Mean computed tomography value to predict the tumor invasiveness in clinical stage IA lung cancer. Ann Thorac Surg.

[6] Matsuguma H, Oki I, Nakahara R, Suzuki H (2013). Comparison of three measurements on computed tomography for the prediction of less invasiveness in patients with clinical stage Ⅰ non-small cell lung cancer. Ann Thorac Surg.

[7] Bierley JD, Gospodarowicz MK, Wittekind C (2017). TNM Classification of Malignant Tumours. 8th ed: Wiley.

[8] Mei XY, Wang R, Yang W (2018). Predicting malignancy of pulmonary ground-glass nodules and their invasiveness by random forest. J Thorac Dis.

[9] Chae HD, Park CM, Park SJ (2014). Computerized texture analysis of persistent part-solid ground-glass nodules: differentiation of preinvasive lesions from invasive pulmonary adenocarcinomas. Radiology.

